# Supplementary material for: Organ specific microenvironmental MR1 expression in cutaneous melanoma
Source: bioRxiv. 2023 Dec 28:2023.12.28.573554. Preprint. [Version 1] doi: 10.1101/2023.12.28.573554 (PMC10836068; doi:10.1101/2023.12.28.573554)
Supplement: Supplement 1 [file NIHPP2023.12.28.573554v1-supplement-1.pdf]

bioRxiv preprint doi: https://doi.org/10.1101/2023.12.28.573554; this version posted December 28, 2023. The copyright holder for this preprint (which was not certified by peer review) is the author/funder. This article is a US Government work. It is not subject to copyright under 17 USC 105 and is also made available for use under a CC0 license.

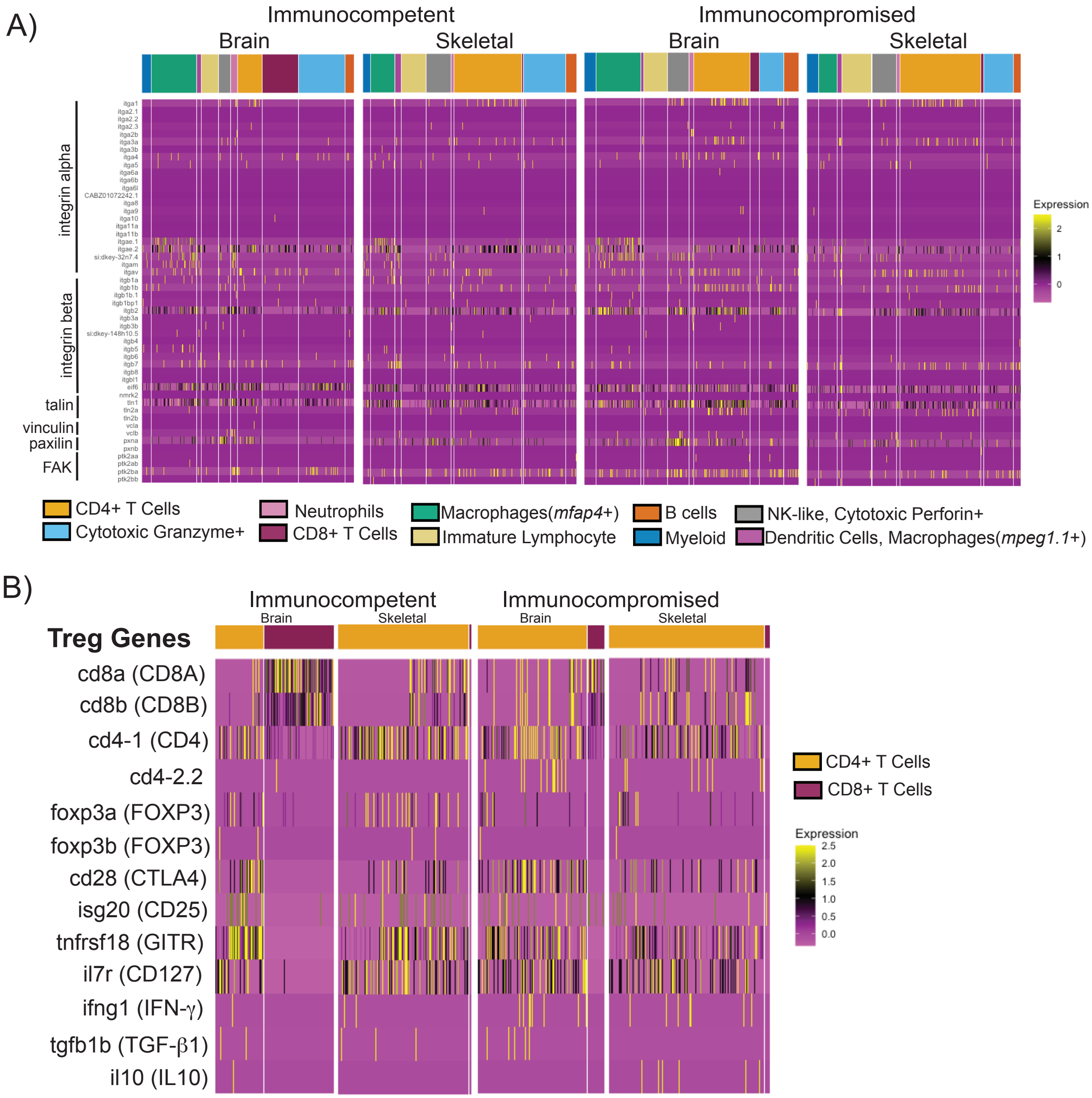

Supplemental Figure 1
(A) Heatmap of alpha and beta integrins and focal adhesion transcripts in immune cell clusters, separated by immune background and organ. (B) Heatmap of several gene transcripts of genes associated with Treg cells of CD4+ and CD8+ T cells separated by immune background and organ.

bioRxiv preprint doi: https://doi.org/10.1101/2023.12.28.573554; this version posted December 28, 2023. The copyright holder for this preprint (which was not certified by peer review) is the author/funder. This article is a US Government work. It is not subject to copyright under 17 USC 105 and is also made available for use under a CC0 license.

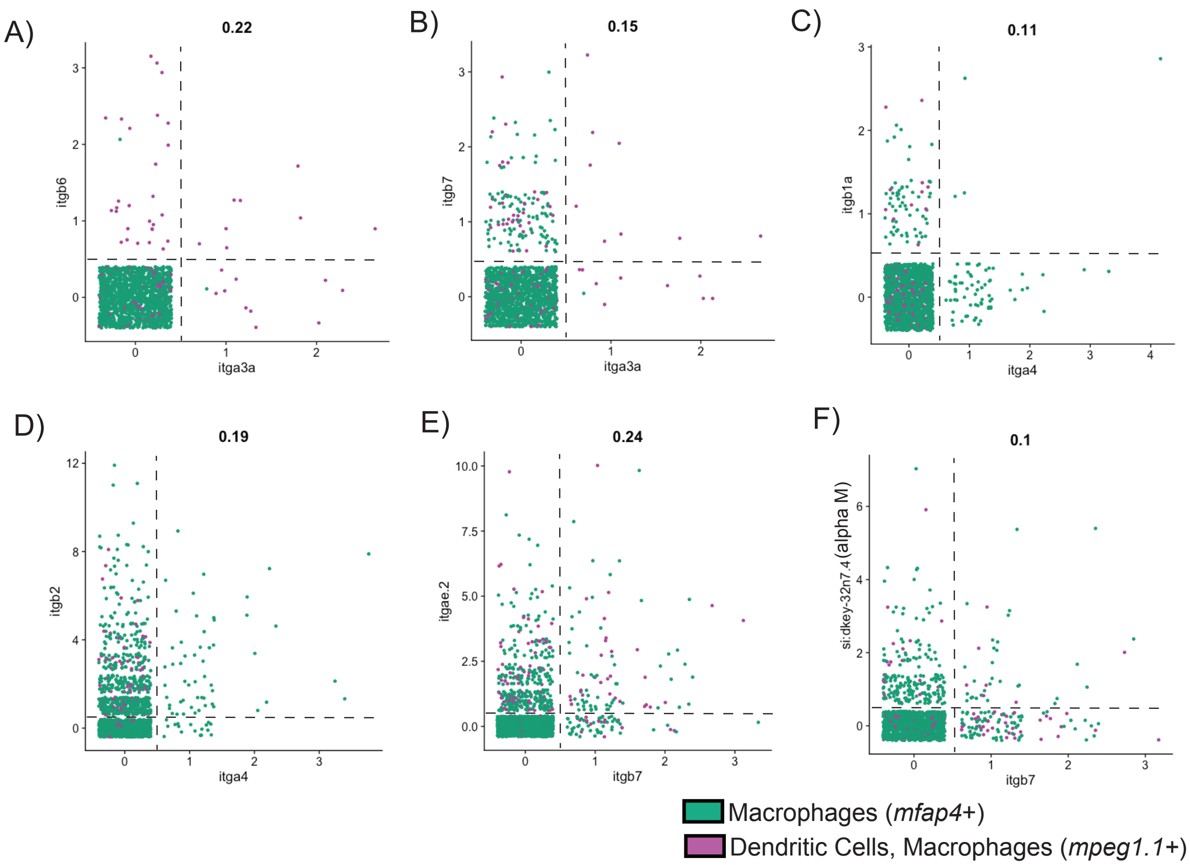

Supplemental Figure 2

(A) Transcript correlation of integrins alpha 3 and beta 6 from macrophage cells (B) Transcript correlation of integrins alpha 3 and beta 7 from macrophage cells (C) Transcript correlation of integrins alpha 4 and beta 1 from macrophage cells (D) Transcript correlation of integrins alpha 4 and beta 2 from macrophage cells (E) Transcript correlation of integrins beta 7 and alpha E from macrophage cells (F) Transcript correlation of integrins beta 7 and alpha M from macrophage cells

bioRxiv preprint doi: https://doi.org/10.1101/2023.12.28.573554; this version posted December 28, 2023. The copyright holder for this preprint (which was not certified by peer review) is the author/funder. This article is a US Government work. It is not subject to copyright under 17 USC 105 and is also made available for use under a CC0 license.

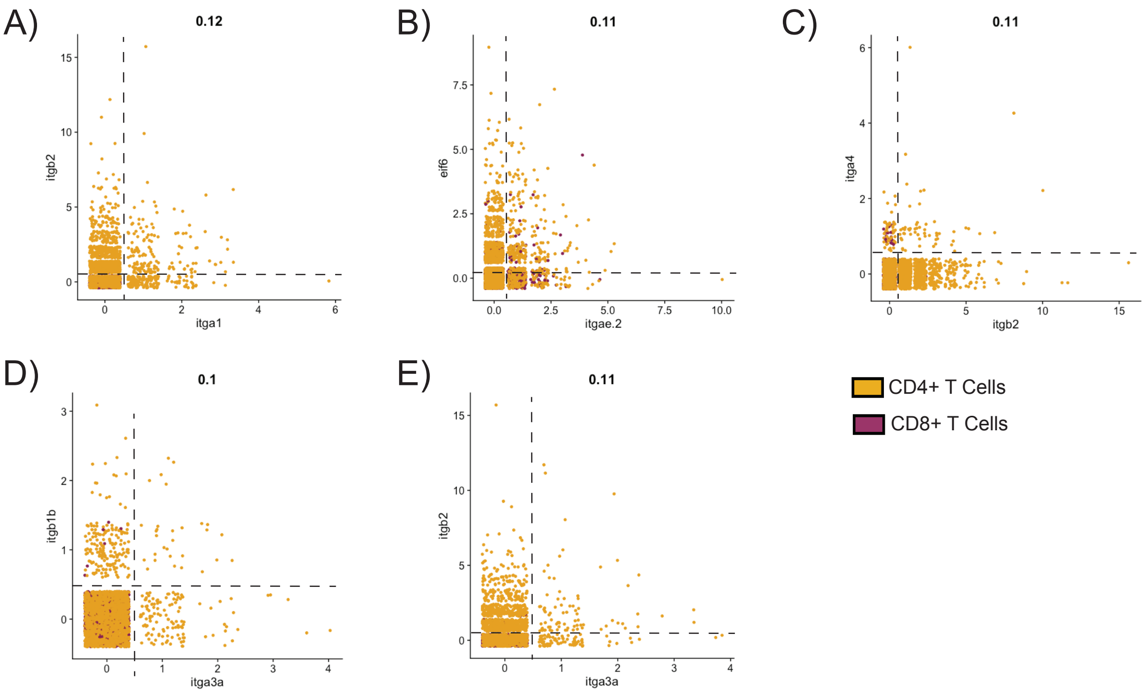

Supplemental Figure 3

(A) Transcript correlation of integrins alpha 1 and beta 2 from T cells (B) Transcript correlation of integrins alpha E and beta 4 binding protein from T cells (C) Transcript correlation of integrins beta 2 and alpha 4 from T cells (D) Transcript correlation of integrins alpha 3 and beta 1 from T cells (E) Transcript correlation of integrins alpha 3 and beta 2 from T cells

bioRxiv preprint doi: https://doi.org/10.1101/2023.12.28.573554; this version posted December 28, 2023. The copyright holder for this preprint (which was not certified by peer review) is the author/funder. This article is a US Government work. It is not subject to copyright under 17 USC 105 and is also made available for use under a CC0 license.

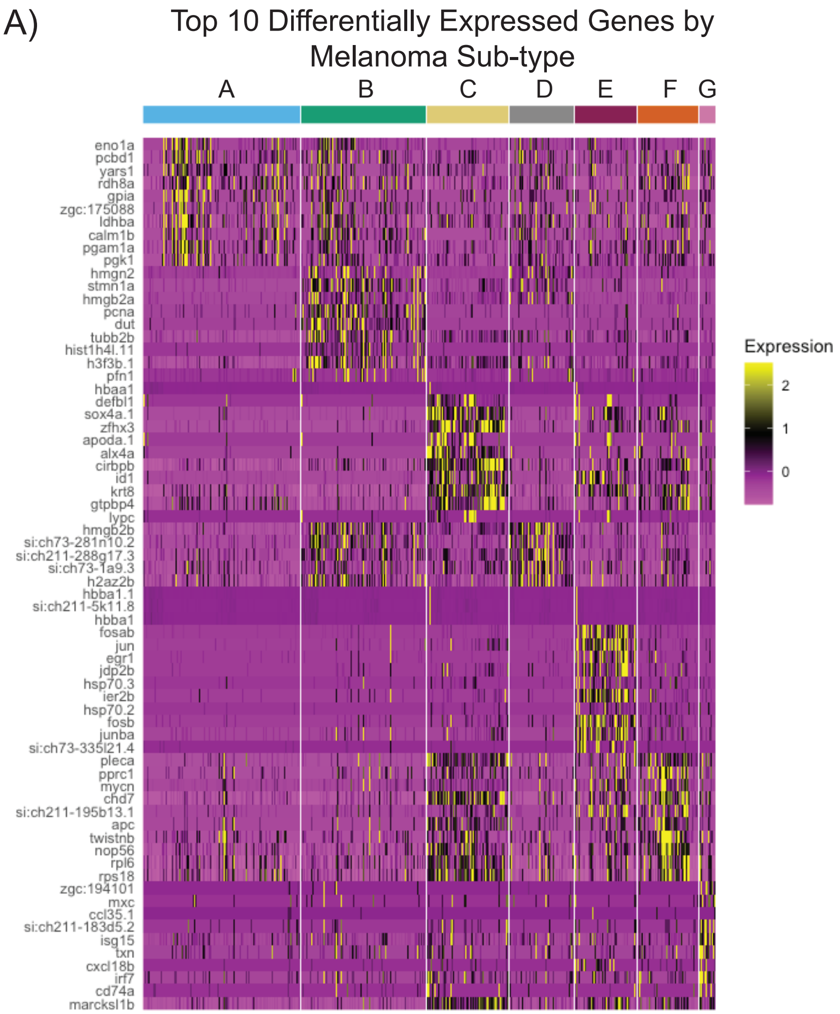

Supplemental Figure 4
(A) Heatmap of the top 10 differentially expressed gene transcripts separated by cluster.

bioRxiv preprint doi: https://doi.org/10.1101/2023.12.28.573554; this version posted December 28, 2023. The copyright holder for this preprint (which was not certified by peer review) is the author/funder. This article is a US Government work. It is not subject to copyright under 17 USC 105 and is also made available for use under a CC0 license.

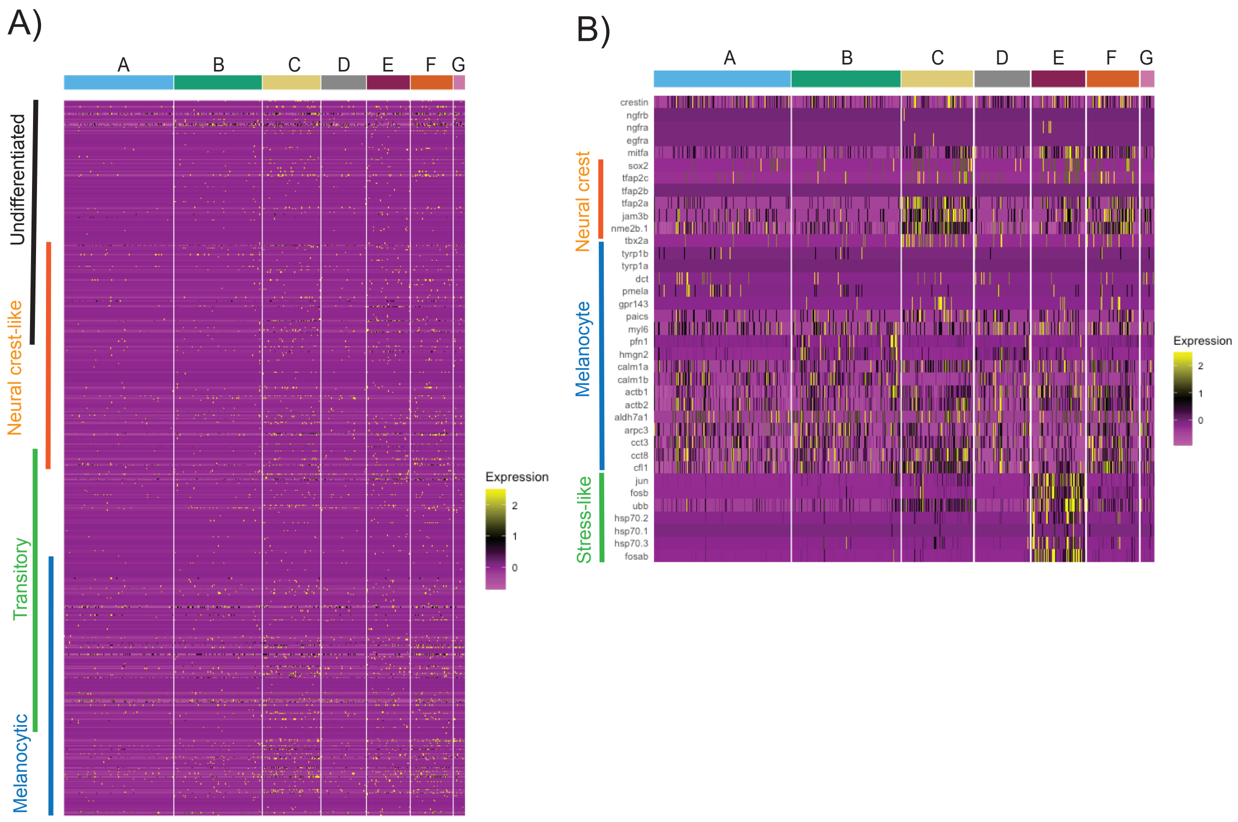

Supplemental Figure 5
(A) Heatmap of gene expression from Tsoi, et al. 2018 melanoma subtype markers (B) Heatmap of gene expression from Baron, et al. 2020 of melanoma subtype markers

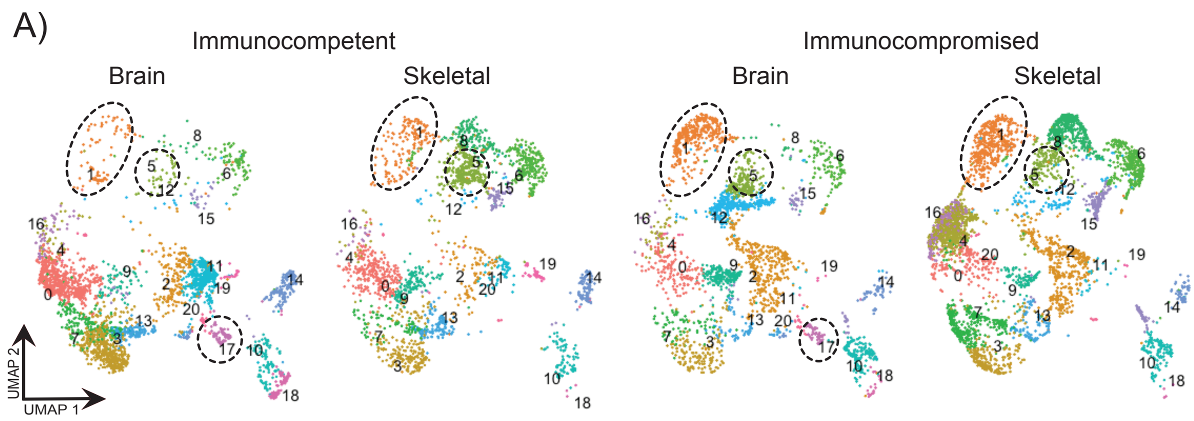

Supplemental Figure 6

bioRxiv preprint doi: https://doi.org/10.1101/2023.12.28.573554; this version posted December 28, 2023. The copyright holder for this preprint (which was not certified by peer review) is the author/funder. This article is a US Government work. It is not subject to copyright under 17 USC 105 and is also made available for use under a CC0 license.

(A) UMAP of original melanoma clusters re-clustered, showing 21 different communities present, notably community 17 is not present in the skeletal samples and communities 1 and 5 are present at a diminished amount in the immunocompetent brain lesion

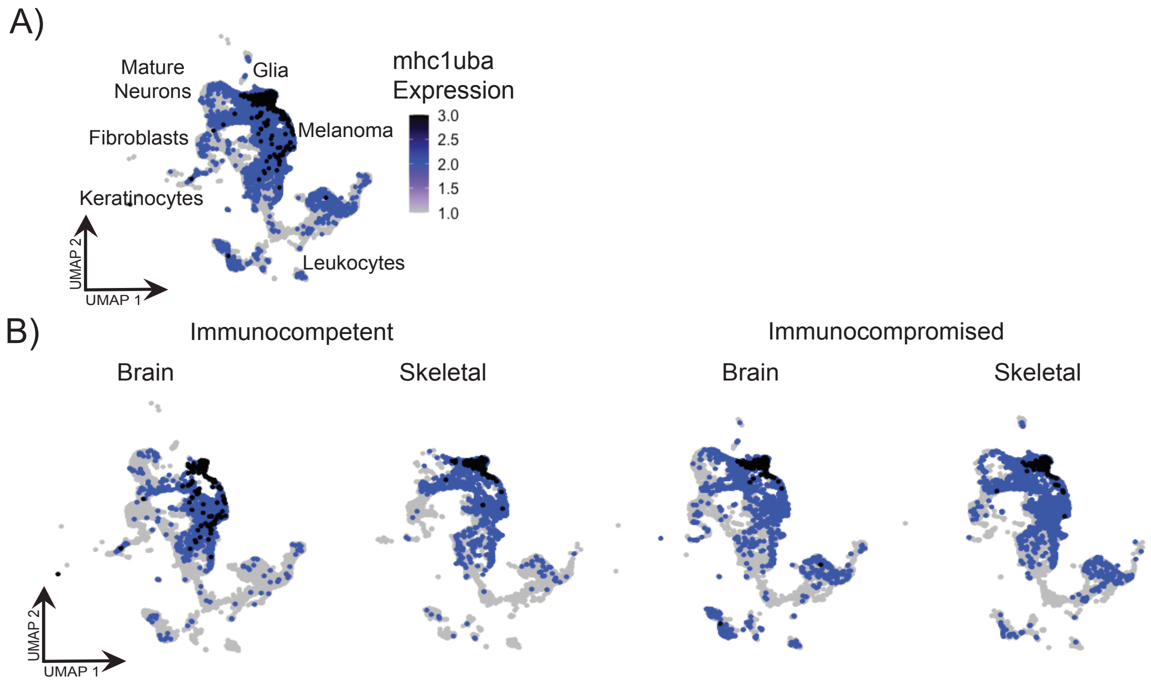

Supplemental Figure 7
(A) UMAP of *mhc1uba* expression of *casper prkdc*+/+ and *casper prkdc*-/- brain and skeletal melanoma samples (B) UMAP of *mhc1uba* expression of *casper prkdc*+/+ and *casper prkdc*-/- brain and skeletal melanoma samples split.

bioRxiv preprint doi: https://doi.org/10.1101/2023.12.28.573554; this version posted December 28, 2023. The copyright holder for this preprint (which was not certified by peer review) is the author/funder. This article is a US Government work. It is not subject to copyright under 17 USC 105 and is also made available for use under a CC0 license.

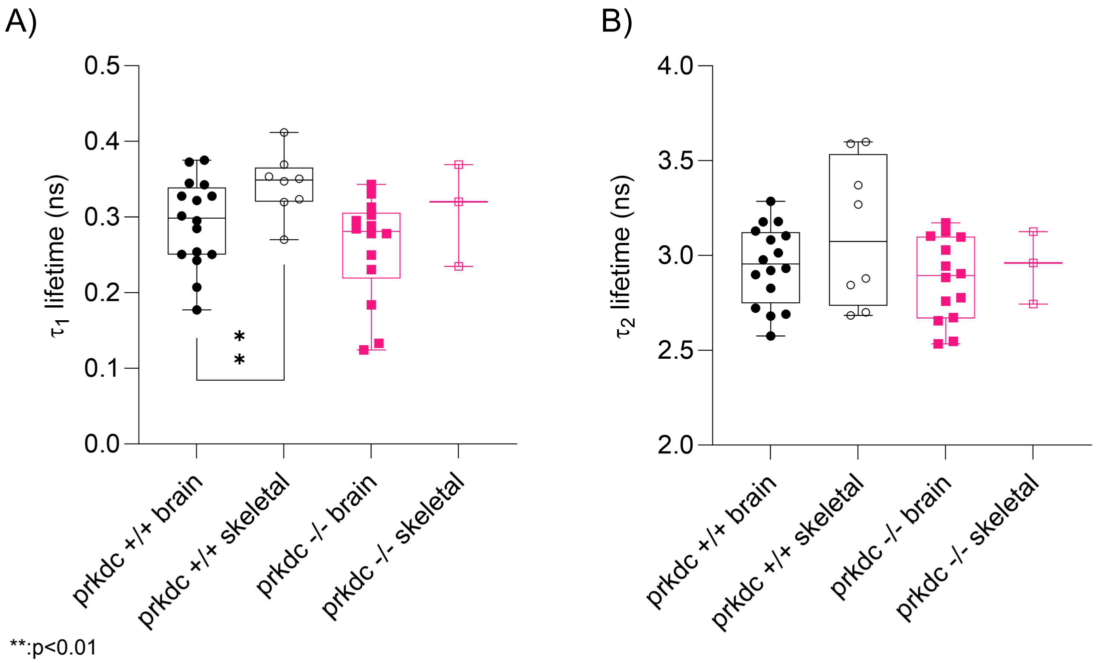

Supplemental Figure 8
(A) lifetime of free NADH (τ_1) for *casper prkdc*+/+ brain (n=15), *casper prkdc*+/+ skeletal (n=8), *casper prkdc*-/- brain (n=13), and *casper prkdc*-/- skeletal (n=3). ** p<0.01, unpaired two-tailed tests. (B) lifetime of bound NADH (τ_2) for *casper prkdc*+/+ brain (n=15), *casper prkdc*+/+ skeletal (n=8), *casper prkdc*-/- brain (n=13), and *casper prkdc*-/- skeletal (n=3).

Tables

Table 1.

**Cell Type**	**Markers (Gene name GRCz11)**
Melanoma	mitfa, crestin, sox10
Erythrocytes	hemgn, cahz, hbba1, hbaa1
Mature Neurons	stmn1b, elavl3, gpm6aa, gap43, ncam1a, ache, nr4a2a, lmx1ba, lmx1bb, gad2, gad1b, slc6a1a, grin1a, slc17a6b
Fibroblasts, Endothelium, Pericytes	pdfgfra, pdgfrb, cola1a, col6a1, col6a2, fn1a, fstl1b, fli1a, kdrl, ccn1, f3b, f3a
Keratinocytes, Epithelium	tp63, krt4, epcam, oclnb
Glia Cells	nes, sox2, tjp1a
CD8+ T cells	lck, cd8a, cd8b, bcl6aa
CD4+ T cells	tox, cd4-1, cd4-2.2, tox2, cd28 (CTLA4), si:dkey-1h24.6 (CD28), pfn1, foxo1a, ptprc
NK-like, Perforin+	nkl.1, nkl.2, nkl.4, gzm3.3, tbx21, prf1.2, prf1.7, prf1.9, arpc1b, foxo1a
Macrophages	mfap4, ctss2.1, ctss2.2, csf1ra, spi1b, marco, prdx5

bioRxiv preprint doi: https://doi.org/10.1101/2023.12.28.573554; this version posted December 28, 2023. The copyright holder for this preprint (which was not certified by peer review) is the author/funder. This article is a US Government work. It is not subject to copyright under 17 USC 105 and is also made available for use under a CC0 license.

Macrophages, Dendritic Cells	cd83, mpeg1.1, mhc2dab, itgam, lcp1
Myeloid Cells	ptprc, mpeg1.1, ctss2.2, spi1b, irf8, mhc2b, lcp1, coro1a
Immature Lymphocytes	nkl.1, pfn1, hmgn3, hmgn2, coro1a, lcp1, arpc1b, rac2, cotl1
Cytotoxic, Granzyme+	gzmk, gata3, tox2
B cells	cd79a, cd79b, ighz
Neutrophils	mpx, mmp9, ncf1

Table 2.

Integrin	Zebrafish Orthologues (Gene name GRCz11)
alpha 1	itga1
alpha 2	itga2.1, itga2.2, itga2.3, itga2b
alpha 3	itga3a, itga3b
alpha 4	itga4
alpha 5	itga5
alpha 6	itga6a, itga6b
alpha 6 like	itga6l
alpha 7	CABZ01072242.1
alpha 8	itga8
alpha 9	itga9
alpha 10	itga10
alpha 11	itga11a, itga11b
alpha E	itgae.1, itgae.2
alpha M	si:dkey-32n7.4, itgam
alpha V	itgav
beta 1	itgb1a, itgb1b, itgb1b.1, itgb1b.2
beta 1 binding protein	itgb1bp1, nmrk2
beta 2	itgb2
beta 3	itgb3a, itgb3b
beta 3 binding protein	si:dkey-148h10.5
beta 4	itgb4
beta 4 binding protein	eif6
beta 5	itgb5
beta 6	itgb6
beta 7	itgb7
beta 8	itgb8
beta like 1	itgbl1
